# 基于分子标志的非小细胞肺癌术后复发预测专家共识

**DOI:** 10.3779/j.issn.1009-3419.2022.102.44

**Published:** 2022-10-20

**Authors:** 广东省胸部疾病学会

**Keywords:** 肺肿瘤, 复发, 分子标志, 共识, Lung neoplasms, Recurrence, Molecular markers, Consensus

## Abstract

近年来，肺癌在筛查、手术、放化疗、靶向治疗以及免疫治疗等各方面都取得了重大进展。目前手术切除仍是局限期非小细胞肺癌(non-small cell lung cancer, NSCLC)最重要的治疗方法，但仍有很多患者在术后5年内出现局部复发或远处转移。目前，NSCLC患者复发风险的评估主要基于临床及病理特征，并不能准确地区分不同复发风险的患者。随着新型检测手段的发展，已发现了多种可能在NSCLC中具有预测复发风险的分子标志。为归纳总结NSCLC患者术后复发相关的分子标志，我们制定了基于分子标志的NSCLC术后复发预测专家共识。共识主要围绕早期NSCLC患者，从分子层面对疾病复发风险进行探讨、归纳和总结。希望能够为早期NSCLC患者提供更多更有价值的预测复发风险工具，从而为疾病管理提供更多帮助。

## Abstract (en)

Significant progress has been made in lung cancer screening, surgery, chemoradiation, targeted therapy, and immunotherapy recently. Surgical resection is the most important treatment for localized non-small cell lung cancer (NSCLC) so far, but there are still many patients who develop local recurrence or distant metastases within 5 years of surgery. Currently, the risk factors of recurrence in patients with NSCLC are mainly based on clinical and pathological features, which hardly identify patients at high risk of recurrence accurately. With the development of new detection technologies, a number of molecular markers that may have a predictive risk of recurrence in NSCLC have been discovered over the years. In order to summarize the molecular markers related to postoperative recurrence in NSCLC patients, we have formulated a consensus on the prediction of postoperative recurrence of NSCLC based on molecular markers. This consensus mainly focuses on the early stage NSCLC patients, discusses and summarizes the risk factors of disease recurrence from the molecular level. It is hoped that more and more valuable information can be provided for the management of patients, so as to provide more guidance for the perioperative management of the patients with early stage NSCLC in the future.

## 1 背景

肺癌是中国当前新发病例和死亡病例最多的肿瘤。据世界卫生组织(World Health Organization, WHO)统计，2020年中国新发肺癌人数82万，肺癌死亡人数71万，均远超其他恶性肿瘤^[1]^。非小细胞肺癌(non-small cell lung cancer, NSCLC)是肺癌的主要类型，约占所有肺癌患者的80%-85%^[2]^。目前手术仍是NSCLC患者首选的治疗方法，只有接受了根治性手术才有彻底治愈的可能。然而NSCLC患者术后复发转移是影响患者预后的关键因素，如何防止术后复发是医生和患者都非常关注的问题。由于NSCLC存在异质性，包括组织学类型、分子特征和驱动基因等，使得患者复发存在较大差异。既往传统的临床研究主要根据病例临床因素(临床分期、吸烟史、性别、组织学类型和手术方式等)作为分析的指标，通过统计学方法对临床特征与复发关系进行分析，得到NSCLC的复发因素，帮助医生对NSCLC患者进行术后复发预测。随着近年来对肺癌生物学理解的加深，NSCLC分子检测取得了重大进展，故临床需要了解肺癌分子机制是如何影响早期可手术NSCLC患者的复发的。鉴于目前缺乏基于肿瘤分子标志信息与术后复发风险的相关内容的总结，我们结合国内相关科室专家的建议及临床证据，制定了基于分子标志的NSCLC术后复发预测共识，希望应用这些新兴的分子标志信息来精确评估NSCLC切除术后的风险，为制定最佳诊疗策略提供重要的理论支持，最终改善早期NSCLC患者预后、提高患者生存率。

本共识使用“resected, early stage, stage I/II/III, lung cancer, lung tumor, risk factors, recurrence, relapse, prognosis”等关键词相互结合检索了PubMed、Web of Science、Google Scholar、EMBASE、中国知网数据库、美国国立综合癌症网络(National Comprehensive Cancer Network, NCCN)指南、中国临床肿瘤学会(Chinese Society of Clinical Oncology, CSCO)指南等，分析了近年来相关文献和研究资料。共识将采用以下推荐级别：1A级：基于高水平证据(样本量较大的研究结果)，专家组有统一认识；1B级：基于高水平证据(样本量较大的研究结果)，专家组无较大争议；2A级：基于低水平证据，专家组有统一认识；2B级：基于低水平证据，专家组无较大争议；3级：不论基于何种级别临床证据，专家组存在较大争议。

## 2 NSCLC驱动基因变异

### 2.1 表皮生长因子受体(epidermal growth factor receptor, *EGFR*)基因突变

*EGFR*突变是NSCLC中最主要的驱动基因变异亚型。该基因的激活性突变可以促进细胞的异常增殖、分化以及血管增生，并能抑制肿瘤细胞的凋亡。癌症基因组图谱(The Cancer Genome Atlas, TCGA)及肿瘤中体细胞突变目录(Catalogue of Somatic Mutations in Cancer, COSMIC)数据显示，*EGFR*在高加索裔的NSCLC患者中的突变率为10%-20%。这一比例在东亚、女性、无吸烟史的肺腺癌患者中尤其高。PIONEER研究^[3]^显示，中国肺腺癌患者*EGFR*突变率达到50%。对于携带*EGFR*突变(最常见突变包括19Del及L858R)的晚期NSCLC患者，已有大量高级别研究^[4-9]^证明其能够从一代到三代EGFR酪氨酸激酶抑制剂(EGFR tyrosine kinase inhibitor, EGFR-TKI)治疗中显著获益，且已成为标准治疗手段。但在可手术患者中，*EGFR*突变是否会增加复发风险、影响总体预后以及不同*EGFR*突变亚型对术后复发风险的影响是否有差异等问题，目前尚无定论。一项来自日本的多中心回顾性研究^[10]^显示，在1, 228例0期-IIIA期手术切除的肺腺癌中，*EGFR*突变型与野生型患者无复发生存(relapse free survival, RFS)并无显著差异。不过该研究也显示，在IIA期-IIIA期以及恶性组织学亚型(包括实性优势型和微乳头亚型)患者中，*EGFR*突变可能提示更差的RFS。而Takamochi等的研究^[11]^显示，在939例手术切除的肺腺癌中(其中Ⅰ期占79%，Ⅱ期-Ⅳ期占21%)，单因素分析显示*EGFR*突变患者具有显著更优的总生存期(overall survival, OS)及RFS，且在多因素分析中，*EGFR*突变被证明是OS的独立的良好预后因子。除此之外，关于不同*EGFR*突变亚型对于预后的影响差异，既往也有相关研究给出了一些初步结论，但这些结论并不一致。上述Takamochi等的研究^[11]^中，418例手术切除的*EGFR*突变肺腺癌患者(Ⅰ期350例，Ⅱ期-Ⅳ期68例)，其中222例(53%)携带*EGFR* L858R，161例(39%)携带*EGFR* 19Del。研究结果显示，不同*EGFR*突变亚型RFS及OS并无差异。而在一项包含4, 872例患者的荟萃分析^[12]^中，研究人员分析了其中1, 020例未接受过EGFR-TKI新辅助或辅助治疗的手术切除的Ⅰ期-Ⅲ期*EGFR*突变NSCLC患者的资料，结果显示*EGFR* 19Del亚组相较于*EGFR* L858R亚组无病生存期(disease free survival, DFS)更差，但并未达到统计学差异。而日本的另一项全国范围内的回顾性研究^[13]^纳入了2, 410例手术切除的*EGFR*突变患者(Ⅰ期1, 629例，Ⅱ期-Ⅳ期765例)，其中1, 170例(48.5%)携带*EGFR* L858R，983例(40.8%)携带*EGFR* 19Del。结果显示，无论是在单变量分析还是多变量分析中，相比于*EGFR* L858R患者，*EGFR* 19Del患者RFS明显更差。

*EGFR*罕见突变在可切除NSCLC患者中的研究目前较少，暂不足以给出建议。

### 2.2 间变性淋巴瘤激酶(anaplastic lymphoma kinase, *ALK*)基因变异

*ALK*重排/融合[常见伴侣包括棘皮动物微管相关蛋白样4(echinoderm microtubule associated protein like 4, *EML4*)、*TFG*、*KIF5B*等]是NSCLC中继*EGFR*突变之后第二个明确可用药的肿瘤驱动基因，在NSCLC中变异比例为2%-7%，多见于不吸烟的年轻肺腺癌患者。*ALK*阳性的转移性NSCLC预后相对较好，目前患者的中位OS可超过5年^[14]^，其主要得益于多款ALK抑制剂显著持久的临床疗效^[15-20]^。众所周知，NSCLC中*ALK*融合以*EML4-ALK*为主，而*EML4-ALK*由于融合位点的不同又可进一步分为多种变体，其中以变异体1和变异体3最为常见^[21-24]^。有研究^[25-27]^进一步指出，*EML4-ALK*融合变异体3(E6; A20)患者使用ALK抑制剂治疗的无进展生存期(progression-free survival, PFS)较短，OS较差。鉴于上述研究，进一步探讨*ALK*融合及不同*EML4-ALK*融合变异体在手术切除NSCLC患者中的预后价值也具有十分重要的临床意义。多项研究^[28-31]^表明*ALK*阳性患者相较于ALK野生型和*EGFR*突变型患者具有更差的RFS和DFS。Yang等^[30]^对300例无吸烟史肺腺癌(其中Ⅰ期58.3%，Ⅱ期5.3%，Ⅲ期20.7%，Ⅳ期15.7%)的研究显示，*ALK*阳性患者5年疾病进展风险是*ALK*阴性患者的2倍以上，且更容易发生脑转移和肝转移。国内的一项单中心回顾性分析^[31]^显示，在675例Ⅰ期-Ⅲ期手术切除的肺腺癌中(其中53.3%接受了辅助治疗)，*ALK*阳性患者相比于ALK野生型患者以及*EGFR*突变患者，均具有显著更短的DFS。Chaft等^[28]^对764例Ⅰ期-Ⅲ期手术切除NSCLC的研究显示，*ALK*阳性患者(29例)较*EGFR*突变患者(255例)具有统计学上更差的RFS。但是需要指出的是，该研究中有41例*EGFR*突变患者接受了辅助TKI治疗，在剔除这部分患者后，*ALK*阳性患者的RFS依然较差，但并无统计学差异。而韩国的一项针对162例IB期-IIIA期无吸烟史肺腺癌的单中心回顾性研究的多因素分析^[29]^显示携带*ALK*或*ROS1*融合的患者具有显著更短的DFS。关于不同*EML4-ALK*变异体对可手术NSCLC患者预后的影响差异，国内有研究发现在可手术患者中*EML4-ALK*融合变异体3(E6; A20)与更差的DFS相关。该研究^[32]^分析了55例Ⅰ期-Ⅲ期手术切除的*ALK*融合肺腺癌患者的数据，多因素分析显示*EML4-ALK*变异体3是更短DFS的独立预后因子。

### 2.3 *KRAS*基因变异

*KRAS*是肺腺癌中的高频突变基因，其热点突变位点按突变频率依次为G12、G13、Q61。*KRAS*在高加索肺腺癌患者中突变频率高达25%，在东亚肺腺癌患者中则低于10%。早期的荟萃分析^[33]^发现*KRAS*突变是腺癌而非鳞癌患者的不良预后因子。该分析纳入的研究包括了Ⅰ期-Ⅳ期患者，在剔除Ⅳ期患者后，结果显示，在Ⅰ期-Ⅲ期患者中*KRAS*对预后的影响不显著。有研究^[34]^纳入了四项临床辅助化疗队列的数据，在1, 543例Ⅰ期-Ⅲ期NSCLC患者中有300例为*KRAS*突变阳性，结果表明*KRAS*外显子12及13的突变与状态不足以预测预后，野生型和突变型患者间DFS和OS均无显著差异。另有研究^[35]^分析了85例*KRAS*突变(其中35例*KRAS* G12C突变患者)、未接受过术前放疗或化疗的手术切除的Ⅰ期-Ⅳ期(其中Ⅰ期-Ⅲ期占96.6%，Ⅳ期占3.4%)肺腺癌患者，多因素分析表明仅*KRAS* G12C是独立的预后预测因子，与明显较短的DFS和OS相关。这一结果在另一项研究中得到了确认，一项纳入230例Ⅰ期-Ⅲ期*KRAS*突变(包括95例*KRAS* G12C)的手术切除肺腺癌患者的研究^[36]^发现，虽然*KRAS*突变与*KRAS*野生型患者3年DFS并无明显差异，但是其中携带*KRAS* G12C突变的患者相比于其他*KRAS*突变类型患者，3年DFS明显更差，且更易出现淋巴血管侵犯和具有较高的肿瘤突变负荷(tumor mutational burden, TMB)。需要注意的是，近期有一项新的荟萃分析^[37]^得出了不同的结论：*KRAS*突变是可切除NSCLC的不良预后因素，携带*KRAS*突变患者具有更差的DFS和OS。此外还有研究^[38]^发现*KRAS* G12V也是可切除肺腺癌的不良预后因素。综上所述，*KRAS*突变可能为NSCLC不良预后因素，且*KRAS* G12C是不良预后因子相对比较明确。

### 2.4 *MET*基因变异

*MET*外显子14跳跃突变或*MET*扩增是NSCLC的已知驱动基因变异。在晚期初治NSCLC中，*MET*外显子14跳跃突变占比约1%-3%(中国患者≤1%，西方患者≤3%)，*MET*扩增占比约1%-2%。而在一代EGFR-TKI耐药后的*EGFR*突变型NSCLC患者中，*MET*扩增占比高达5%-20%；三代EGFR-TKI耐药后，*MET*扩增发生率可能更高。在一项纳入687例NSCLC患者(583例Ⅰ期-IIIA期)的研究^[39]^中发现了18例*MET*外显子14跳跃突变和8例*MET*扩增患者，多因素分析表明*MET*外显子14跳跃突变及*MET*扩增是NSCLC独立的不良预后因素。另一项研究^[40]^得出了类似的结果，447例接受根治性手术切除的NSCLC患者中(Ⅰ期-Ⅲ期占94%，Ⅳ期占6%)检出48例*MET*高拷贝数，其中18例确认为*MET*基因扩增。结果显示，相比于*MET*低拷贝数患者，*MET*高拷贝数患者具有显著更短的OS。多因素分析显示，*MET*拷贝数扩增是手术切除后的一个独立的不良预后因素。但是这些研究都存在样本量太少的问题。近期的一项荟萃分析^[41]^弥补了这个缺陷，该分析共纳入20个队列包括共600例左右的*MET*扩增阳性NSCLC患者(包含Ⅰ期-Ⅳ期)，结果显示高*MET*基因拷贝数扩增是独立不良预后因素，尤其是在腺癌和亚洲患者中。

### 2.5 *ROS1*基因变异

*ROS1*重排/融合被公认为NSCLC重要的分子亚型之一，在NSCLC中发生率约1%-2%。*ROS1*融合伴侣包括*CD74*、*SLC34A2*、*TPM3*、*SDC4*、*EZR*、*KDELR2*、*CCDC6*等。在晚期肺癌中ROS1对患者生存率的影响仍然存在争议。一项大型回顾性研究^[42]^发现淋巴结转移患者的*ROS1*融合阳性率较高。此外有研究^[43]^报道*ROS1*融合阳性状态与微乳头成分和气源性扩散高度相关，后者已被确定为侵袭性肿瘤生物学的标志物。但是也有研究^[44]^发现，在晚期NSCLC中ROS1不仅是ROS1抑制剂的疗效预测因子，更是独立的预后标志物，*ROS1*阳性患者的预后显著优于*ALK*阳性、*EGFR*阳性等其他亚组人群。在可手术NSCLC患者中，既往韩国的一项针对162例IB期-IIIA期无吸烟史肺腺癌的单中心回顾性研究^[29]^中，多因素分析显示携带*ALK*或*ROS1*融合的患者具有显著更短的DFS。考虑到ROS1对NSCLC术后预后影响的研究较少，因此暂时不应作为术后预后因素。

### 2.6 *BRAF*基因变异

*BRAF*在肺腺癌、肺鳞癌中突变频率分别约为8%、5%，多见于吸烟患者。*BRAF*突变通常分布在整个外显子11和15中，其中大多数突变发生在编码激酶催化结构域的外显子15上。根据信号传导机制、激酶活性和对抑制剂的敏感性，*BRAF*突变可被分为三个类别：RAS非依赖性激酶激活V600单体(I类)、RAS非依赖性激酶激活二聚体(II类)和RAS依赖性激酶失活异二聚体(III类)^[45, 46]^。I类突变是最主要的突变类型，占所有*BRAF*突变的20%-30%，常见*BRAF* V600D/E/K/R突变，导致非依赖RAS激活BRAF激酶活性，进一步激活MAPK/ERK途径，对BRAF和MEK抑制剂敏感。II类突变包括*BRAF* K601、L597、G464和G469突变，约占所有*BRAF*突变的20%。这些突变位于活化片段或位点，是RAS非依赖性二聚体，可能不会从EGFR靶向药物治疗中获益。III类包括*BRAF* G466、N581、D594和D596突变，这些基因突变位于结合位点折叠的催化环或DGF基序，激酶活性低于野生型BRAF或缺乏激酶活性，可能从EGFR靶向药物治疗中获益。研究^[46, 47]^显示对于晚期NSCLC，I类突变的预后似乎略好于II类和III类突变，后两者与更具侵袭性的肿瘤行为和更差的临床状况以及一线化疗后的疾病进展较快有关。但是目前尚缺乏足够的证据明确*BRAF*突变对NSCLC预后的影响，尤其是在早期可手术患者中。

### 2.7 *RET*基因变异

NSCLC中≤1%的患者携带*RET*重排/融合，且几乎全部为肺腺癌。*RET*重排/融合为明确的驱动基因变异，其最常见的融合伴侣是*KIF5B*，第二个常见的融合伴侣是*CCDC6*，其他融合伴侣包括*NCOA4*、*EPHA5*和*PICALM*等。一项纳入5, 800余例美国NSCLC患者的研究^[48]^显示，在调整基线协变量后，在选择性RET抑制剂可用之前，*RET*重排/融合对患者的OS或PFS没有影响。一项荟萃分析^[49]^共纳入13项研究，包括121例阳性*KIF5B-RET*重排/融合的患者，结果显示*KIF5B-RET*融合基因阳性和阴性患者的OS和PFS无差异，其中早期(Ⅰ期及Ⅱ期)患者同样未显示出差异。同样，考虑到*RET*融合对NSCLC术后预后影响的研究较少，因此暂时不应作为术后预后因素。

### 2.8 TP53

编码p53蛋白的肿瘤抑制基因*TP53*的突变在NSCLC的所有亚型中都很常见。据报道^[50, 51]^，腺癌的*TP53*突变率为39%-46%，鳞状细胞癌为81%，大细胞癌为68%。*TP53*通过细胞周期阻滞、凋亡、衰老或控制代谢和DNA修复，在预防和抑制异常细胞生长中发挥多重作用。研究^[52]^显示，*TP53*非断裂性突变(non-disruptive mutation)是晚期NSCLC(包括*EGFR*突变型)患者的不良预后因子。2016年的一项纳入19项研究的荟萃分析^[53]^结果显示，在3, 371例Ⅰ期-Ⅳ期NSCLC中，将*TP53*突变组(*n*=1, 406)与野生型组(*n*=1, 965)进行比较，野生型组的总生存率显著高于突变型组；亚组分析发现，在临床早期(Ⅰ期/Ⅱ期)NSCLC患者亚组以及腺癌病理亚型组中都有着类似的结果，而非腺癌及临床晚期患者中*TP53*突变与OS并无显著相关性。另一项前瞻性研究^[54]^还表明，在245例Ⅰ期-IIIA期手术切除的NSCLC中，*TP53*突变可预测Ⅰ期患者的较低的4年生存率，但在Ⅱ期-IIIA期患者中则不然。另外Chien等^[55]^研究中的Ⅰ期*TP53*野生型NSCLC患者与*TP53*突变患者相比，中位生存时间有更长的趋势，但未达到显著差异；而在Ⅱ期-Ⅲ期患者却未见此现象，反而有相反趋势。而Ma等^[56]^对LACE-Bio项目中的四项辅助化疗临床研究(IALT、JBR.10、CALGB 9633和ANITA)的1, 209例Ⅰ期-Ⅲ期手术切除的NSCLC患者的数据进行分析发现，在总人群中*TP53*突变对预后无影响；但对于*TP53*野生型患者，接受辅助化疗后具有更优的DFS和OS，而*TP53*突变患者不能从辅助化疗中获益。这一现象在鳞癌中更明显。上述差异可能是未对*TP53*具体突变类型进一步分析造成的。众所周知，并非所有*TP53*突变都具有相同的临床意义，因此可能需要通过区分具体*TP53*突变亚型来考虑其对预后的影响^[57, 58]^。Jiao等^[59]^首先根据变异情况将*TP53*分为四组：A组为野生型；B组包括外显子2、3和10的变异；C组包括外显子5、7、8和9的变异(预后良好)；D组包括外显子4、6的变异以及多外显子突变和未知外显子突变(预后不良)。但是该分组的结果尚未在其他队列中得到验证。另一种分类为两分类：破坏性突变及非破坏性突变。但不同研究有不同的结果^[52, 59, 60]^。因此，*TP53*突变的具体分类及在可手术NSCLC患者中的具体临床意义还需要进一步区分和明确。

**共识一**

***EGFR*常见突变中，与携带*EGFR* L858R突变的患者相比，携带*EGFR* 19Del的患者术后复发风险更高(2A级)。**

***ALK*重排/融合阳性患者术后预后更差，尤其是携带*EML4-ALK*融合变异体3(E6; A20)的患者(2A级)。**

***KRAS*突变为潜在的NSCLC不良预后因素(2B级)，其中*KRAS* G12C突变在肺腺癌中是相对较明确的复发高危因素(2A级)。**

***MET*扩增是NSCLC术后的独立不良预后因素(1A级)，小样本研究提示*MET*外显子14跳跃突变同样是不良预后因素(2A级)。**

***TP53*突变可能是NSCLC术后不良预后因素，且不能从辅助化疗中获益(2B级)。**

## 3 免疫相关分子标志

### 3.1 TMB

TMB一般指特定基因组区域内每兆碱基对(Mb)体细胞非同义突变的个数。TMB最初被提出是作为肿瘤细胞中由突变蛋白产生的新抗原数量的标志物，后者可由细胞表面上的主要组织相容性复合体(major histocompatibility complex, MHC)分子呈递。通常认为具有更多新抗原产生的肿瘤更可能在细胞表面上表达被识别为非自身的新抗原，并且在免疫治疗剂存在下引发更强的CD8^+^ T细胞应答，因此TMB常被视为免疫治疗疗效的预测因子。当然，新抗原形成相当复杂且难以预测，不一定与TMB绝对相关^[61]^。目前在NSCLC中已经有相当多的程序性死亡受体1(programmed cell death 1, PD-1)/程序性死亡配体1(programmed cell death ligand 1, PD-L1)抑制剂，临床试验证实TMB可以作为免疫治疗疗效的预测因子。一线Nivolumab治疗NSCLC的CheckMate 026试验的亚组分析^[62]^显示，TMB状态以及PD-L1表达水平是疗效的预测性生物标志物；而KEYNOTE-158研究^[63]^也显示高TMB患者更能从免疫治疗中获益。TMB在晚期NSCLC中作为免疫治疗效果的预测因子已经得到明确的证实并被纳入指南^[64]^。但是目前的大多数研究都聚焦于TMB对免疫治疗疗效的预测，关于TMB本身对NSCLC患者尤其是可手术患者的预测复发风险的研究相对偏少而且相互矛盾。Devarakonda等的研究^[65]^纳入了908例来自LACE-Bio-II研究的可切除NSCLC患者的数据(Ⅰ期-Ⅱ期占87.5%，Ⅲ期-Ⅳ期占12.5%)，其结果显示1515基因大panel检测出的高TMB(> 8个突变/Mb)与切除术后患者更长的OS、DFS以及肺癌特异性生存(lung cancer specific survival, LCSS)显著相关，提示TMB可作为术后复发风险分层因素。除此之外，研究人员还发现，高TMB患者接受辅助化疗后并未延长其LCSS；相反，低TMB(≤4个突变/Mb)患者接受辅助化疗后能够延长LCSS。日本的一项研究^[66]^利用全外显子组测序(whole exome sequencing, WES)技术对90例手术切除的IA期-IIIB期NSCLC患者(均未接受过术前化疗或免疫治疗)进行TMB检测，结果显示患者中位TMB为62个。然而，多因素分析发现高TMB(即TMB > 62个突变)患者OS显著更差(HR=12.31, *P*=0.019)；而在Ⅰ期NSCLC患者中，高TMB与较差的OS(HR=7.582, *P*=0.001, 8)和DFS(HR=6.07, *P*=0.007, 2)相关。而国内研究人员开展的一项针对TCGA数据库中454例高加索人群肺腺癌(82.6%为早中期，17.4%为晚期)的研究数据^[67]^显示，无论是早期还是晚期肺腺癌，TMB状态均与预后无关；该研究还分析了49例中国晚期肺腺癌患者TMB与预后的关系，结果同样显示TMB状态与预后无关。但是需要指出的是，上述研究TMB检测覆盖基因组区域及cut-off值均不同，患者的临床治疗情况存在一定差别，这些因素可能是造成研究结论不同的原因。此外，需要注意的是TMB尤其是组织TMB(tissue TMB, tTMB)检测很难通过一次检测描述整个肿瘤的突变负荷情况。研究^[68]^表明在通过多区域活检评估4例肾细胞癌患者的体细胞突变情况时，一次活检只能检测到肿瘤内所有突变的55%；此外，各次活检检出的相同突变仅占所有突变的34%，表明大多数突变无法通过单次活检检测到。导致单次TMB检测无法完整描述整个肿瘤的突变负荷情况的主要原因是肿瘤内异质性(intratumoral heterogeneity, ITH)，因此补充评估ITH的情况也可能给予更好的预后判断。2014年在*Science*上发表的一项纳入11例Ⅰ期-Ⅲ期肺腺癌患者的研究^[69]^对患者肿瘤组织的多个区域分别进行WES检测，结果显示，患者原发灶中亚克隆突变占比较高与术后复发风险增加相关。2017年发表在*The New England Journal of Medicine*的TRACERx研究^[70]^分享了其对100例IA期-Ⅲ期NSCLC患者肿瘤多区域全外显子检测结果：亚克隆拷贝数异常比例高(异常比例≥48%)意味着体细胞拷贝数异质性更高，患者复发或死亡风险高于亚克隆拷贝数异常比例低(< 48%)的患者(HR=4.9, *P*=4.4×10^-4^)；即使在调整年龄、吸烟、分期等因素后，差异依然显著(HR=3.70, *P*=0.01)。2020年另一项纳入84例肺腺癌手术患者(Ⅰ期-Ⅲ期占97.6%，Ⅳ期占2.4%)的研究^[71]^发现，亚克隆拷贝数异常比例高的患者的OS较比例低的患者更差，这与TRACERx研究的结果类似。需要指出的是这些研究中亚克隆基因突变或拷贝数异常占比的计算方法及cut-off值均不统一。

### 3.2 PD-1/PD-L1

针对PD-1和PD-L1的免疫检查点抑制剂为NSCLC患者提供了新的治疗机会。PD-1及其配体(PD-L1, PD-L2)在抑制NSCLC患者的免疫系统中起主要作用。PD-1在免疫细胞[T细胞、B细胞、自然杀伤(natural killer, NK)细胞、树突细胞和单核细胞]上表达，而PD-L1主要在肿瘤细胞上表达。目前针对PD-1的抗体(Nivolumab及Pembrolizumab)或PD-L1抗体(Atezolizumab、Durvalumab和Avelumab)的两类药物均在晚期NSCLC患者的Ⅲ期临床试验中显示出相应的疗效^[72-76]^。而目前随着Impower010研究的成功，辅助免疫治疗也逐渐开始成为临床新的关注点。在可手术的NSCLC患者中PD-1/PD-L1的表达水平和预后的关系也逐渐为人们所关注。关于PD-L1表达水平在可手术患者中与预后关系的研究相对较多，其中具有代表性的是2017年发表在*Annals of Oncology*上的一项研究。该研究^[77]^入组了LACE-Bio项目中的三项临床研究(IALT、JBR.10和CALGB 9633)的982例Ⅰ期-Ⅲ期可手术切除的NSCLC患者，通过免疫组织化学(E1L3N抗体)评估患者肿瘤细胞及免疫细胞膜上PD-L1的蛋白表达状态，使用不同的cut-off值(≥1%、≥10%、≥25%和≥50%)分析PD-L1表达情况与预后的关系以及PD-L1表达是否能够预测辅助化疗疗效。结果显示，无论肿瘤细胞或免疫细胞膜表面的PD-L1表达水平如何，其既不是预后因素，也不是辅助化疗疗效的预测因子。Kim等^[78]^对331例Ⅰ期-Ⅲ期肺鳞癌的研究也得到了类似结论。当然，关于PD-L1的正向预后预测价值也有不少相关研究。例如，Cooper等^[79]^的研究纳入了678例Ⅰ期-Ⅲ期可手术的NSCLC患者，多因素分析显示肿瘤细胞PD-L1高表达(≥50%)与更长的OS显著相关。Zaric等^[80]^对161例Ⅰ期-Ⅲ期完全切除肺腺癌患者的研究也表明，肿瘤细胞PD-L1阳性相比于PD-L1阴性患者具有显著延长的OS。Yang等^[81]^对163例手术切除的Ⅰ期肺腺癌患者的研究显示，肿瘤细胞PD-L1表达水平虽与OS无关，但是PD-L1阳性患者具有显著更高的5年RFS率。另一项在321例Ⅰ期-Ⅲ期根治性切除的NSCLC中的研究^[82]^表明，虽然肿瘤细胞PD-L1表达与整个研究队列的OS无关，但是在鳞癌、接受过辅助治疗、肿瘤体积较大(pT2-4)、淋巴结阳性(pN1-3)这四类特定人群中，PD-L1阳性提示显著更优的OS。与之相类似的结论，即PD-L1表达在部分特定亚组人群中具有预后提示价值，也在其他一些研究^[83-85]^中被报道。值得注意的是，另外一些研究^[86, 87]^则得出了相反的结论，即PD-L1高表达是不良预后因素。例如：Azuma等^[86]^的研究表明，在164例IA期-IIIB期NSCLC手术患者中，采用二分法划分的PD-L1高表达患者相比于低表达患者具有显著更短的OS。Zhang等^[87]^对143例Ⅰ期-Ⅲ期的可手术肺腺癌的研究也得到了类似结论：PD-L1阳性人群具有显著更差的RFS和OS。

关于免疫细胞PD-1表达水平与预后相关性的研究则相对较少。Kim等^[78]^对331例Ⅰ期-Ⅲ期肺鳞癌的研究表明，PD-1^+^肿瘤浸润淋巴细胞(tumor infiltrating lymphocytes, TILs)数量增加与显著更长的OS相关。除此之外，Paulsen等^[85]^对536例手术切除的Ⅰ期-IIIA期NSCLC的研究表明，鳞癌亚型中更高密度的PD-1^+^ TILs与更好的疾病特异性生存(disease specific survival, DSS)相关，而腺癌中无此相关性。值得注意的是，也有研究显示PD-1^+^ TILs能够预测腺癌亚型的OS。例如，Zaric等^[80]^纳入161例Ⅰ期-Ⅲ期完全切除的肺腺癌患者，多因素分析显示，免疫细胞PD-1^+^是RFS及OS的独立预后因子。Cooper等^[79]^、Schmidt等^[82]^研究也表明，虽然PD-1^+^ TILs在321例Ⅰ期-Ⅲ期根治性切除的NSCLC队列中不是显著的预后因素，但是在非鳞癌亚组中，PD-1^+^ TILs与更长的OS相关。

与TMB类似，在可手术的NSCLC中，虽然已有报道表明PD-1/PD-L1具有预后预测价值，但同时也存在与之相矛盾的结论。而且，各研究中所用的PD-1/PD-L1检测抗体、平台以及判定标准尚不统一。因此，目前尚无法明确PD-1/PD-L1作为预测复发风险标志物的临床价值。

### 3.3 肿瘤微环境(tumor microenvironment, TME)

TME即肿瘤细胞产生和生存的内环境，存在大量与肿瘤细胞密切联系的非肿瘤细胞、组织及活性分子，对于肿瘤的发生、生长和转移起着重要作用^[88]^。其在不同癌种甚至不同患者中存在着较大差异，Lambrechts等^[89]^利用单细胞测序技术描绘了肺癌的TME图谱，在原发非转移患者的肿瘤病灶中鉴别出了52个间质细胞亚群，主要包括免疫细胞(髓细胞、T细胞和B细胞)、成纤维细胞、内皮细胞、肺泡细胞和上皮细胞。肿瘤细胞和TME中的非肿瘤成分紧密交互形成了一个复杂的动态网络结构，针对其复杂性和异质性，随着技术的发展，对于TME的研究手段也在不断更新，主要包括免疫组化(immunohistochemistry, IHC)、多重免疫荧光(multiplex immunofluorescence, mIF)、流式细胞(flow cytometry, FACS)、RNA测序、单细胞RNA测序(single-cell RNA-seq, ScRNA-seq)、WES等技术。TME中的免疫细胞通过免疫编辑影响着癌细胞的生长和进化，故在临床上尤为受到关注。Federico等^[90]^在最新研究中利用高维度FACS技术并根据TILs类型将局限性NSCLC患者分为IM1和IM2两个亚型。IM2型与不良预后相关，患者肿瘤重存在表达CD103、PD-1、T细胞免疫球蛋白粘蛋白3(T cell immunoglobulin and mucin domain 3, TIM-3)和可诱导协同刺激分子(inducible costimulate, ICOS)的TILs，与之相反，IM1型患者则有显著更长的RFS，肿瘤病灶聚集大量表达细胞溶解酶的CD8^+^ T细胞，缺乏抑制性受体的CD4^+^ T细胞(特别是CD103^+^、TIM-3^-^、PD-1^-^)及B细胞浸润和三级淋巴结构(tertiary lymphoid structure, TLS)水平增加。T淋巴细胞作为功能亚群最多且与肿瘤杀伤、免疫逃逸等机制直接相关的免疫细胞，既往临床研究^[91]^表明，CD45RO^+^、CD3^+^、CD8^+^ T细胞在临床上普遍与NSCLC预后呈正相关。一项纳入797例Ⅰ期-IIIA期的NSCLC患者的研究^[92]^发现，肿瘤间质的CD8^+^ T细胞不仅是DFS和OS的独立预后因素，且可作为除肿瘤原发灶-淋巴结-转移(tumor-node-metastasis, TNM)分期之外的额外预后因子。联合PD-1的表达情况，Mazzaschi等的研究^[93]^报道在手术治疗的NSCLC患者队列中PD-1/CD8比值不论是单因素还是多因素分析均显示其可作为OS的独立预测因子，且低比值的患者OS更长。Li等^[94]^通过分析既往发表的转录组数据发现，DNA损伤修复基因*APE1*与初始CD4^+^ T细胞浸润呈负相关，在纳入108例手术患者的队列中IHC实验证实APE1的表达与CD4^+^ T细胞浸润亦呈负相关，且有CD4^+^ T细胞高浸润的患者仅在APE1低表达时与更长的RFS相关。调节性T细胞(Treg)通常被认为起着负向调节机体免疫反应的作用，研究^[95, 96]^表明对于接受手术治疗的NSCLC患者FOXP3^+^ Treg细胞的高浸润(实质和间质)与不良OS、RFS相关。2018年，Guo等^[97]^利用单细胞测序构建可手术NSCLC患者的T细胞图谱的研究进一步证实肿瘤激活Treg相关的基因标签表达在腺癌中与更差的预后相关。而一项纳入80例可手术NSCLC患者的研究^[98]^则得出了相反的结论，该研究发现肿瘤浸润的CD4^+^和CD8^+^ T细胞与OS及DFS均不相关，但肿瘤间质高浸润的CD4^+^FOXP3^+^ Treg细胞则与更长的OS显著相关。肿瘤相关巨噬细胞(tumor associated macrophage, TAM)主要分为经典激活的巨噬细胞(M1型)和交替激活的巨噬细胞(M2型)。一项纳入663例Ⅰ期-Ⅲ期可手术NSCLC患者的研究^[99]^显示，高浸润的(瘤内和间质)HLA-DR^+^CD68^+^ M1型、CD204^+^ M2型、CD68^+^ TAM与更好的预后相关且M1型是独立的正向预后因子。但亦有研究^[100, 101]^报道CD68^+^CD163^+^ M2型TAM高浸润患者有更短的OS。TLS是指在肿瘤中存在的异位淋巴组织，有关肺鳞癌的研究^[102]^表明(队列里0期-IIIA期占90%)，TLS密度可以作为与PFS相关的独立正向预后因子。Rakaee等^[103]^基于可手术NSCLC患者的研究得出类似的结论，且建立的TLS score评分系统可以有效地改善病理分期的预后能力。目前，鉴于TME的复杂性和异质性，对于早期可手术的NSCLC患者，尚无统一的TME亚型或细胞成分作为可手术患者的预测复发风险的因子。

### 3.4 肿瘤新抗原负荷(tumor neoantigen burden, TNB)

TNB是反映肿瘤细胞中总的新生抗原数量的一个指标，通常以每百万碱基(Mb)肿瘤基因组区包含的肿瘤新生抗原数量来表示，现阶段可作为TMB的补充评估指标。肿瘤产生的非同义突变编码形成的蛋白会被蛋白酶降解成8个-11个氨基酸肽段，经过与MHC结合后被递呈至肿瘤细胞表面，被T细胞识别后激活免疫反应。目前多项研究^[104-106]^表明TNB在多个癌种中与肿瘤免疫治疗疗效及预后相关。McGranahan等^[107]^通过分析来自TCGA队列的Ⅰ期-Ⅳ期NSCLC患者生存数据发现，在肺腺癌中高TNB的患者OS显著高于低TNB的患者，而在肺鳞癌人群中却未发现差异，分析其原因可能与MHC-1表达水平低有关。后续发表的另一项仅针对于肺鳞癌的研究^[108]^同样发现TNB水平与预后无相关性。除此之外，既往研究^[109, 110]^表明ITH水平可作为评估预后以及免疫治疗疗效的指标，ITH水平低的患者更有可能从免疫治疗中获益，这与ITH水平低的患者存在较高比例的主克隆新生抗原且这部分新生抗原与MHC分子具有较高的亲和力有关。因此当结合ITH水平评估TNB时研究发现主克隆性新抗原数量相比总新抗原数量能更好地反映免疫治疗获益^[111]^，但目前TNB作为肺癌术后复发风险的预测因子暂无相关报道。

**共识二**

**基于目前证据，TMB、PD-L1尚不足以作为NSCLC术后的预后因素(2A级)。**

**肿瘤中亚克隆基因突变或拷贝数异常占比较高的NSCLC患者术后复发风险较高(2A级)。**

**目前研究显示，TME相关免疫细胞可能与NSCLC患者术后复发相关，但尚需更多研究以明确其预测复发的意义(2B级)。**

**TNB可能为肺腺癌预后因素，但尚缺乏其作为NSCLC术后复发风险预测因子的证据(3级)。**

## 4 肺癌多基因检测

多基因表达预测模型目前已应用于NSCLC患者术后的复发风险分层。肺癌14基因检测(朗迪瑞^TM^)是全球第一个用于鉴别Ⅰ期-IIA期非鳞NSCLC患者术后复发风险且辅助指导术后辅助化疗的检测产品。其专有算法整合了14个基因(*BAG1*、*BRCA1*、*CDC6*、*CDK2AP1*、*ERBB3*、*FUT3*、*IL-11*、*LCK*、*RND3*、*SH3BGR*、*WNT3A*、*ESD*、*TBP*、*YAP1*)的mRNA表达水平，并在多项独立研究^[112-115]^中得到性能验证。有研究^[96]^发现，在330例NCCN指南标准评估为低复发风险的IA期-IB期患者中，仍有部分患者被肺癌14基因检测识别为分子中/高风险，其预后明显差于分子低风险患者。同时在269例TNM分期为IA1期患者中，有部分患者仍可被肺癌14基因检测定义为分子中/高风险，其预后也明显差于分子低风险患者^[97]^。这两项研究提示NCCN指南风险分层及TNM分期对高复发风险患者的识别具有局限性，而肺癌14基因检测对早期非鳞NSCLC具有更好的复发风险分层作用。肺癌14基因检测在中国人群中开展的大规模回顾性研究^[96]^表明，在1, 006例Ⅰ期-Ⅲ期中国早期R0切除非鳞NSCLC患者的队列中，无论是总体队列还是各分期亚组，随着肺癌14基因检测风险分层风险等级的上升，患者的5年生存率明显降低。一项纳入100例Ⅰ期-IIA期非鳞NSCLC患者的前瞻性研究结果^[114]^显示，肺癌14基因检测作为预测因子，可预测早期非鳞NSCLC患者术后复发风险：在不接受辅助化疗的前提下，被肺癌14基因检测识别为低风险的IA期-IIA期患者5年DFS率达93.8%，而中/高风险的患者5年DFS率仅为58.8%。此外，肺癌14基因检测能鉴别出从辅助化疗中获益的患者。研究^[114]^显示肺癌14基因检测识别的中/高风险患者采用铂类辅助化疗的5年DFS率为91.7%，而未采用化疗的中/高风险患者的5年DFS率仅为48.9%。在随后的一项纳入250例Ⅰ期-IIA期(主要是IA期患者，177例)非鳞NSCLC患者的前瞻性研究^[115]^中，肺癌14基因检测风险分层对术后复发风险以及辅助化疗获益的预测作用再次得到了验证：低风险患者的5年无复发(freedom from recurrence, FFR)率达到94.6%，未接受辅助化疗的中/高风险患者5年FFR率为72.4%，接受辅助化疗的中/高风险患者5年FFR率则可达到97.0%；同时，无论*EGFR*为野生型还是突变型，肺癌14基因检测仍可识别能够从辅助化疗中获益的中/高风险患者。遗憾的是，目前尚无可预测肺鳞癌术后复发风险的相关产品，期待将来能够有相关检测产品应用于临床。

**共识三**

**多个预后基因的表达可以整合用于肺癌患者术后的复发风险分层；推荐IA期-IIA期R0切除的非鳞NSCLC进行肺癌14基因(*BAG1*、*BRCA1*、*CDC6*、*CDK2AP1*、*ERBB3*、*FUT3*、*IL-11*、*LCK*、*RND3*、*SH3BGR*、*WNT3A*、*ESD*、*TBP*、*YAP1*)的mRNA表达检测，无论*EGFR*阳性或阴性患者，肺癌14基因检测均可识别出能够从辅助化疗中获益的中/高风险患者(1A级)。**

## 5 微小残留病灶(minimal residual disease, MRD)

MRD是指经过治疗后，传统影像学[包括正电子发射计算机断层显像(positron emission tomography/computed tomography, PET/CT)]或实验室方法不能发现，但通过液体活检发现的癌来源分子异常，代表着肺癌的持续存在和潜在的临床进展可能。TRACERx研究^[116]^对24例早期NSCLC患者术后通过Signatera检测追踪外周血中的个体化单核苷酸变异(single nucleotide variant, SNV)，其监测SNV中位数为18个，范围10个-22个，结果显示在14例ctDNA阳性(MRD阳性，至少检出2个SNV)的患者中13例患者出现临床疾病复发，且ctDNA阳性较影像学复发提前时间为70天(范围10天-346天)。随后TRACERx研究进一步公布了其后续78例病例结果，通过ArcherDx(中位196个SNV，范围72个-482个)动态监测ctDNA，结果显示45例疾病复发患者中37例ctDNA阳性，较影像学/临床复发提前预判时间为151天；而另外23例无复发患者中的199份血浆样本中仅检测出1份ctDNA阳性^[117]^。另一项研究^[118]^通过CAPP-Seq技术动态追踪40例Ⅰ期-Ⅲ期接受根治性治疗的NSCLC患者，结果提示其中20例任一时间节点的ctDNA阳性患者全部出现疾病复发，其提前预判时间为5.2个月。因此，从目前研究数据看，基于术后ctDNA的MRD检测在预测早期NSCLC患者术后疾病复发预测与监控方面已初露锋芒。现有报道的MRD检测中位最大丰度分布在0.1%-0.01%之间，提示MRD检测时，ctDNA检测的敏感性需要触达万分位的数量级，所以敏感性是MRD检测的最大瓶颈。因此在MRD检测阴性的情况下，可能需要结合其他分子标志来综合评判NSCLC患者术后复发风险情况。目前使用甲基化技术应用于MRD检测的产品为Guardant Health公司的Guardant Reveal，该产品可同时检测ctDNA突变及ctDNA甲基化，即基于血浆cfDNA的基因组和表观基因组信息。目前已获美国纽约州卫生部临床实验室评估计划(Clinical Laboratory Evaluation Program, CLEP)批准用于检测和监测早期结直肠癌患者的MRD。但该产品在肺癌中尚缺乏充分研究数据。同时，利用甲基化信息是否能够帮助预测肿瘤复发部位，目前尚无相关报道。在第18届中国肺癌高峰论坛上已达成了我国首个《非小细胞肺癌分子残留病灶专家共识》，对NSCLC MRD的概念、基本技术要求、临床应用已有较明晰的指导意见^[119]^。但目前不同研究之间关于MRD的定义及研究方法差别较大，研究结论之间难以完全互通，并且国内MRD检测产品尚缺乏足够证据，还需进一步探索及积累数据。

**共识四**

**早期NSCLC患者行根治性切除术后，MRD阳性提示复发风险高，需进行更密切的随访管理并考虑更积极的干预策略。但MRD检测方法及评定标准需要规范化，其稳定敏感性还有待提升。在MRD检测阴性的情况下，建议结合其他分子标志来综合评判NSCLC患者术后复发风险情况(2A级)。**

## 6 基于临床病理因素构建的预后预测模型

除上述基于分子标志物预测NSCLC术后复发风险相关性的研究外，Liang等^[120]^国内学者基于临床病理因素构建的可切除NSCLC生存列线图(nomogram)在预后预测方面也具有重要的临床应用价值。该研究分析了来自国内多个中心的5, 261例接受根治术的NSCLC患者的资料，并鉴定出年龄、性别、组织学类型、T分期、N分期、淋巴结采样站数6个独立预后因素，并根据各因素对预后的影响大小按权重分配评分以建立列线图。内部验证结果显示，该模型预测患者的1年、3年及5年生存率和实际观察到的生存情况高度吻合，且其对OS的预测效能比美国癌症联合委员会(American Joint Committee on Cancer, AJCC)第七版TNM分期更优。研究人员又利用国际肺癌研究协会(International Association for the Study of Lung Cancer, IASLC)数据库中的2, 148例患者对此模型进行了独立外部验证，进一步证实了该模型的临床价值。值得一提的是，该研究团队后续又利用监测、流行病学和最终结果(Surveillance, Epidemiology, and End Results, SEER)数据库中30, 475例NSCLC患者资料，鉴定出年龄、性别、淋巴结采样站数、肿瘤大小、手术范围、肿瘤分化程度、组织学类型和是否胸膜浸润8个LCSS率的独立预后因素，并由此构建了第八版TNM Ⅰ期NSCLC特有的生存预测模型，并通过中国多中心的1, 133例患者队列对此模型进行了独立验证^[121]^。上述研究表明，单纯基于临床病理因素构建的预后预测模型也具有重要的临床应用价值，并且与分子标志物的预后预测具有相互独立的价值。

目前WHO肺肿瘤分类将原位腺癌(adenocarcinoma *in situ*, AIS)和微浸润性腺癌(minimally invasive adenocarcinoma, MIA)定义为没有或有限的组织学侵袭性成分的癌症，研究^[122, 123]^发现MIA和AIS患者的生存率没有显著差异，并且AIS和MIA患者术后5年无复发的概率为100%。鉴于浸润前病变(AIS和MIA)患者术后无需给予辅助治疗便可获得良好预后，因此此类患者无需进行相应分子检测预测术后复发。

**共识五**

**基于临床病理高危因素构建的预后预测模型也具有重要预测价值，且与分子标志物、驱动基因的预后价值相互独立(2A级)。**

**浸润前病变(AIS和MIA)及其他预后好的患者，术后可不进行相应分子检测(2A级)。**

## 7 总结

早期肺癌是一种可通过手术治疗实现治愈的恶性肿瘤。尽管早期NSCLC患者整体预后较好，但大约30%的早期NSCLC患者在术后5年内复发^[124]^。针对该问题，本共识首次归纳总结与NSCLC根治术后患者复发、转移密切相关的分子标志，为针对性地采取干预措施，从而降低复发和转移事件的发生提供参考。

**Table:** 共识专家组成员

**共识指导专家**		**蒋峰**	**江苏省肿瘤医院**	**史加海**	**南通大学附属医院**	
**何建行**	**广州医科大学附属第一医院**	**蒋鹏**	**兰州大学第二医院**	**宋平平**	**山东省肿瘤医院**	
**执笔组长**		**矫文捷**	**青岛大学附属医院**	**孙大强**	**天津市胸科医院**	
**梁文华**	**广州医科大学附属第一医院**	**金龙玉**	**中南大学湘雅三医院**	**田辉**	**山东大学齐鲁医院**	
**执笔委员会成员(按姓名拼音排序)**		**雷光焰**	**陕西省肿瘤医院**	**田子强**	**河北医科大学第四医院**	
**阿迪力**	**新疆肿瘤医院**	**雷艺炎**	**中山大学附属第一医院**	**王光锁**	**深圳市人民医院**	
**巴玉峰**	**河南省肿瘤医院**	**李斌**	**兰州大学第二医院**	**王宏涛**	**陕西省人民医院**	
**蔡开灿**	**南方医科大学南方医院**	**李鹤飞**	**河北大学附属医院**	**王洪琰**	**河北医科大学第四医院**	
**蔡林波**	**广东三九脑科医院**	**李辉**	**南方医科大学珠江医院**	**王继勇**	**广州中医药大学第一附属医院**	
**陈椿**	**福建医科大学协和医院**	**李少民**	**西安交通大学第二附属医院**	**王文祥**	**湖南省肿瘤医院**	
**陈海泉**	**复旦大学附属肿瘤医院**	**李树本**	**广州医科大学附属第一医院**	**魏立**	**河南省人民医院**	
**陈炬**	**中山大学孙逸仙纪念医院**	**李伟**	**潍坊市人民医院**	**温昭科**	**广西壮族自治区人民医院**	
**陈军**	**天津医科大学总医院**	**李向楠**	**郑州大学第一附属医院**	**吴蔚**	**陆军军医大学第一附属医院**	
**陈奇勋**	**浙江省肿瘤医院**	**李小强**	**北京大学深圳医院**	**夏振坤**	**中南大学湘雅二医院**	
**成向阳**	**广州医科大学附属第一医院**	**李新举**	**西安交通大学第一附属医院**	**谢德耀**	**温州医科大学附属第一医院**	
**程超**	**中山大学附属第一医院**	**梁乃新**	**北京协和医院**	**谢展鸿**	**广州医科大学附属第一医院**	
**范江**	**上海市第一人民医院**	**梁胜景**	**广西壮族自治区人民医院**	**熊刚**	**南方医科大学南方医院**	
**方文涛**	**上海市胸科医院**	**梁文华**	**广州医科大学附属第一医院**	**徐世东**	**哈尔滨医科大学附属肿瘤医院**	
**付军科**	**西安交通大学第一附属医院**	**廖洪映**	**中山大学附属第六医院**	**许顺**	**中国医科大学附属第一医院**	
**高阳**	**中南大学湘雅医院**	**廖虎**	**四川大学华西上锦医院**	**薛涛**	**东南大学附属中大医院**	
**葛棣**	**复旦大学附属中山医院**	**廖永德**	**武汉协和医院**	**杨林**	**深圳市人民医院**	
**耿国军**	**厦门大学附属第一医院**	**刘德若**	**北京中日友好医院**	**杨懿**	**成都市第三人民医院**	
**耿庆**	**武汉大学人民医院**	**刘宏旭**	**辽宁省肿瘤医院**	**殷伟强**	**广州医科大学附属第一医院**	
**古卫权**	**佛山市第一人民医院**	**刘继先**	**北京大学深圳医院**	**岳东升**	**天津医科大学肿瘤医院**	
**郭晓彤**	**中国医学科学院肿瘤医院深圳医院**	**刘伟**	**吉林大学白求恩第一医院**	**张毅**	**首都医科大学宣武医院**	
**韩彪**	**兰州大学第一医院**	**刘阳**	**中国人民解放军总医院**	**张志辉**	**湛江中心医院**	
**韩育宁**	**宁夏医科大学总院**	**刘洋**	**四川省肿瘤医院**	**赵国芳**	**宁波华美医院**	
**何建行**	**广州医科大学附属第一医院**	**刘长宏**	**大连医科大学第二附属医院**	**赵健**	**广州医科大学附属肿瘤医院**	
**何明**	**河北省第四医院**	**罗猛**	**贵州省人民医院**	**赵军**	**苏州大学附属第一医院**	
**何苡**	**河南省人民医院**	**马海涛**	**苏州大学附属第一医院**	**赵松**	**郑州大学第一附属医院**	
**赫捷**	**中国医学科学院肿瘤医院**	**毛广显**	**北京大学深圳医院**	**赵晓菁**	**上海交通大学医学院附属仁济医院**	
**胡鸿**	**复旦大学附属肿瘤医院**	**潘华光**	**安徽医科大学第一附属医院**	**周承志**	**广州医科大学附属第一医院**	
**胡坚**	**浙江大学附属第一医院**	**潘小杰**	**福建省立医院**	**周建平**	**东莞市人民医院**	
**胡牧**	**北京友谊医院**	**彭忠民**	**山东省立医院**	**周清华**	**四川大学华西医院**	
**黄海涛**	**苏州大学附属第一医院**	**乔贵宾**	**广东省人民医院**	**朱余明**	**上海市肺科医院**	
**姜涛**	**空军军医大学唐都医院**	**任胜祥**	**上海市肺科医院**			
